# Paraspeckles Are Associated with the Activation and Nuclear Localization of Unphosphorylated miR-34a

**DOI:** 10.3390/ncrna12020012

**Published:** 2026-03-31

**Authors:** Graham H. Read, Kristen McGreevy, Hanny Issawi, Tiffany Yang, Cynthia Tsang, Ihsan A. Turk, Emily Rietdorf, Whitaker Cohn, David W. Salzman, Julian P. Whitelegge, Joanne B. Weidhaas

**Affiliations:** 1 Radiation Oncology Department, University of California, Los Angeles, CA 90095, USA; grahamhread@gmail.com (G.H.R.); tyang2001@g.ucla.edu (T.Y.);; 2 Biostatistics, MiraDx, Los Angeles, CA 90025, USA; 3 Department of Biostatistics, University of California, Los Angeles, CA 90095, USA; 4 Pasarow Mass Spectrometry Lab, University of California, Los Angeles, CA 90095, USA; jwhitelegge@mednet.ucla.edu

**Keywords:** radiation, microRNA, paraspeckle, miR-34a

## Abstract

**Background/Objectives:** Canonical microRNAs possess a 5′ phosphate required for Argonaute binding and activity. However, prior work identified an unphosphorylated, inactive nuclear pool of the important radiation-responsive microRNA, miR-34, that is rapidly phosphorylated and activated in response to ionizing radiation (IR). Here, we extend this work and investigate the role of paraspeckles, a phase-separated nuclear sub-compartment, and their association with the localization of unphosphorylated miR-34a. **Methods:** Mass spectrometry was performed to identify interacting partners of unphosphorylated mir-34. CRISPR-mediated deletion of the paraspeckle NEAT1_2 triple helix motif was performed to create an A549 cell line lacking paraspeckles (dTH). Activity and expression of mir-34a post-irradiation were evaluated by qRT-PCR and luciferase assays comparing dTH and wild-type (WT) A549 cell lines. In situ hybridization (ISH) was performed to evaluate mir-34a localization before and after IR, comparing dTH and WT cell lines. **Results:** Mass spectrometry identified paraspeckle proteins as significantly enriched interacting partners of unphosphorylated mir-34 mimics. By qRT-PCR and luciferase assays, we found that paraspeckle loss prevented radiation-induced early activation of unphosphorylated mir-34a. We found no difference in radiation-induced transcription of pri-miR-34a, but early processing to pre-miR-34a appeared delayed. ISH confirmed that loss of paraspeckles altered the nuclear localization of miR-34a before and after IR. **Conclusions:** These data suggest that paraspeckles are associated with nuclear localization and early radiation-responsive activation of unphosphorylated miR-34a. This suggests a coordinated nuclear sequestration of this important miR in its unphosphorylated state to enable an enhanced radiation response.

## 1. Introduction

Canonical microRNA (miRNA) regulation is a two-step process occurring in multiple cellular compartments—miRNAs are transcribed as long, hairpinned transcripts, processed by the Microprocessor complex, exported to the cytoplasm, and processed again by Dicer to generate a ~22–25 nucleotide-long transcript with a 5′ phosphate [1,2]. This 5′ phosphate is required for binding to Argonaute (Ago2), which is required both for stabilizing the miRNA and directing it to target mRNAs [3].

The tumor suppressor miRNA miR-34 has been implicated in several key pathways in cancer cells, including metastasis, apoptosis, cell cycle regulation, and DNA repair [4,5]. To this end, miR-34 is well characterized as a tumor suppressor [6,7], with higher miR-34 expression correlating with improved patient survival [8] and increasing interest in miR-34 delivery as a therapeutic [6,9,10]. Ionizing radiation (IR) induces de novo miR-34 expression in a p53-dependent manner [11]; accordingly, miR-34 has been well described as an important determinant of cancer cell radiosensitivity [5,12].

Uniquely among miRNAs, the miR-34 family has been shown to maintain a fully processed unphosphorylated pool in the nucleus [13]. This pool is rapidly phosphorylated after IR, causing an increase in miR-34 activity prior to de novo transcription and processing [13]. While the existence and activation of unphosphorylated miR-34 have been characterized, mechanisms governing the nuclear localization, stabilization, and protection of unphosphorylated miR-34 have not previously been defined.

Paraspeckles are a membrane-less, phase-separated sub-compartment in the nucleus formed by several critical proteins associated with the long noncoding RNA (lncRNA) NEAT1_2 [14], which is stabilized by a triple helix motif in lieu of polyadenylation [15,16,17]. Paraspeckles maintain a distinct, stimulus-responsive cargo separate from the rest of the nucleoplasm [18,19]. Accordingly, paraspeckles have been implicated in several stress response pathways for their ability to sequester specific factors and coordinate their colocalization in response to stressors [20,21,22], and NEAT1_2 has been shown to sponge several miRNA species in an Ago2-dependent manner, including miR-34 [23,24]. Interestingly, NEAT1_2 also contains a pseudo-pri-miRNA sequence that has been shown to recruit DROSHA, implicating paraspeckles in the processing of a select pool of pri-miRNAs [25]. Accordingly, several works have described NEAT1_2 as a potential cancer biomarker, often in the context of its ability to sponge other RNA species [26,27,28].

This study investigates the possible role of paraspeckles in the nuclear localization and stabilization of the unphosphorylated, radiation-responsive miR-34a pool. We find that unphosphorylated miR-34a interacts with nuclear paraspeckle proteins, and then by creating cells without paraspeckles through deletion of NEAT1, we evaluate the impact of paraspeckle loss on radiation-responsive activity of miR-34a, the levels of phosphorylated miR-34a at early time points after IR, as well as the presence of punctate nuclear populations of miR-34 prior to radiation. While our findings do not define the mechanisms by which paraspeckles interact with miR-34a, they support the hypothesis that paraspeckles are associated with the stability and localization of unphosphorylated miR-34a prior to radiation.

## 2. Results

### 2.1. Paraspeckle Proteins Interact with Unphosphorylated miR-34a

**Figure 1:**
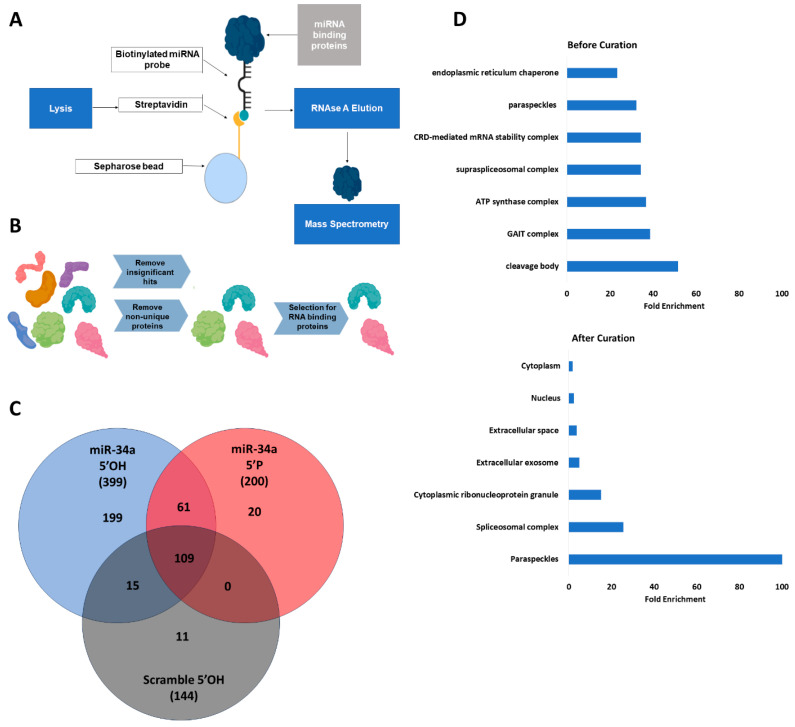
Mass spectrometry identifies nuclear paraspeckles as interacting partners of unphosphorylated miR-34 probes. (**A**) Workflow of sample isolation for mass spectrometry. Streptavidin sepharose beads were incubated with biotinylated miRNA mimics and mixed with sonicated whole-cell lysates. Samples were eluted by RNAse A treatment. The figure was created with Biorender. (**B**) Curation of mass spectrometry hits. Candidate proteins were identified by significance, uniqueness to the 5′OH miR-34a probe, and presence of known RNA-binding activity. (**C**) Characterization of isolated proteins. The total number of proteins isolated binding to the indicated probe is shown in parentheses, with the number of proteins unique to that sample or overlapping with other samples shown in the Venn diagram. (**D**) Gene ontology (GO) curation for cellular compartments performed on proteins identified in the 5′OH miR-34a eluates before and after curation (which was the removal of hits with a score below 20, *p* value > 0.05, non-unique proteins across three replicates, and non-RNA-binding proteins).

Since rates of miR-34a transcription in A549s are low at baseline [29] and the unphosphorylated pool is detectably large [13], we hypothesized that unphosphorylated miR-34a must be stabilized and protected somewhere within cells. We performed streptavidin-biotin affinity purification to identify candidate proteins interacting uniquely with 5′OH miR-34a mimics, and not with 5′P miR-34a or 5′OH miRNA mimics with scrambled sequences [30]. A schematic of this mass spectrometry workflow can be found in Figure 1A.

Candidate proteins were identified as those unique to the 5′OH miR-34a sample, present in three independent replicates, and expressing a canonical RNA-binding domain (Figure 1B) [31]. We identified 199 proteins unique to the 5′OH miR-34a sample and confirmed specific binding via affinity purification (Supplemental Figure S1A). Proteins unique to the 5′OH miR-34a pulldown represent 49.8 percent of all proteins identified in affinity-purified lysates (Figure 1C). Overlap between samples was common—109 proteins were detected binding to all RNA probes, and 61 bound to any miR-34a mimic independent of 5′ phosphorylation status. Gene ontology (GO) analysis [32,33] on cellular compartments for significant proteins unique to the 5′OH miR-34a eluates showed strong enrichment of nuclear paraspeckle proteins, leading to the hypothesis that paraspeckles interact with 5′OH miR-34a (Figure 1D). This set represented 52.5% of all categorized paraspeckle proteins and 85.7% of all proteins required for paraspeckle formation [34].

### 2.2. Cas9-Mediated Deletion of NEAT1 to Eliminate Paraspeckle Formation

**Figure 2:**
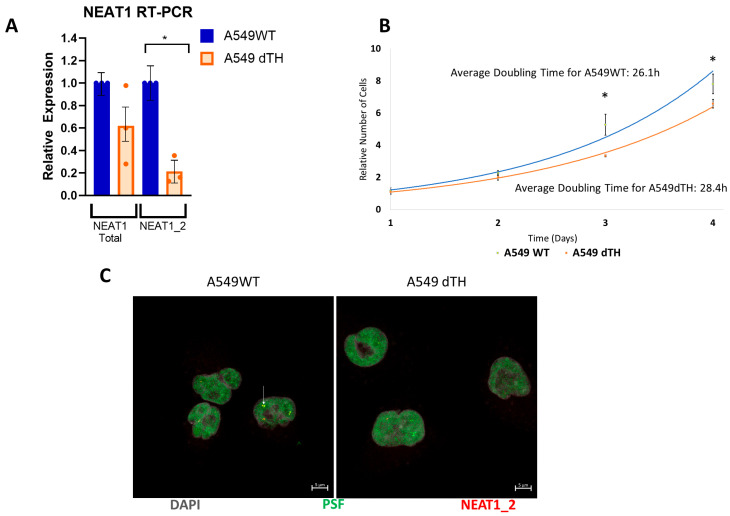
Characterization of a Cas9-mediated deletion of the NEAT1_2 triple helix domain. (**A**) qRT-PCR against sequences in NEAT1_1 (NEAT1 Total) and NEAT1_2 in wild-type and triple helix-deleted cells. * *p* = 0.034 by one sample *t*-test. (**B**) Cell counts over time of cultured wild-type and dTH cells. Doubling time was calculated by exponential regression. * *p* = 0.005 by two-sample *t*-test at 3 days and *p* = 0.127 at 4 days. (**C**) Paired immunofluorescence and in situ hybridization for the paraspeckle marker protein PSF and a region in the middle of NEAT1_2. An example of a colocalized focus is indicated with white arrows.

Based on the apparent interaction between paraspeckle proteins and unphosphorylated miR-34, we chose to investigate the effect of paraspeckle loss on unphosphorylated mir-34a. We thus designed a Cas9-mediated deletion of the triple helix motif of the lncRNA NEAT1_2 (NEAT1 deletion of the triple helix [dTH]) spanning from the stem-loop motif and past the 3′ terminus of the transcript, slightly larger than a design previously reported to prevent paraspeckle formation [15]. qRT-PCR for both NEAT1_2 and the shorter, triple helix-independent isoform NEAT1_1 revealed a significant decrease in NEAT1_2 levels (one sample *t*-test *p* = 0.034). While trending downwards, we did not find a significant difference in total NEAT1 expression (one-sample *t*-test *p* = 0.240), confirming selective destabilization of the longer isoform (Figure 2A). Consistent with prior reports that loss of NEAT1_2 is phenotypically mild [15,35], dTH cells showed no morphological differences and no difference in viability or significant differences in the rate of growth (Figure 2B). In situ hybridization for middle regions of NEAT1_2 alongside immunofluorescence for the paraspeckle marker protein PSF showed colocalization in wild-type A549 cells, with punctate signal for both targets lost upon deletion of the NEAT1_2 triple helix motif in dTH cells (Figure 2C), confirming loss of paraspeckle formation in these cells.

### 2.3. Paraspeckles Appear Necessary for Early Radiation-Responsive miR-34a Activity

To assess the activity of radiation-responsive miR-34a, previously created dual-luciferase reporters [13] (Promega, Madison, WI, USA) were used to quantify relative capacity for miRNAs to bind to repressive 3′UTR elements [13]. These data were paired with qRT-PCR for mature miRNA sequences to assess the relative expression of miRNAs after IR. Since prior work demonstrated that early miR-34a activity after IR originates from phosphorylation of the unphosphorylated miR-34a pool [13], we hypothesized that loss of paraspeckles might eliminate this unphosphorylated pool or potentially lead to the loss of phosphorylation of this pool, resulting in the loss of early miR-34a activity after IR.

**Figure 3:**
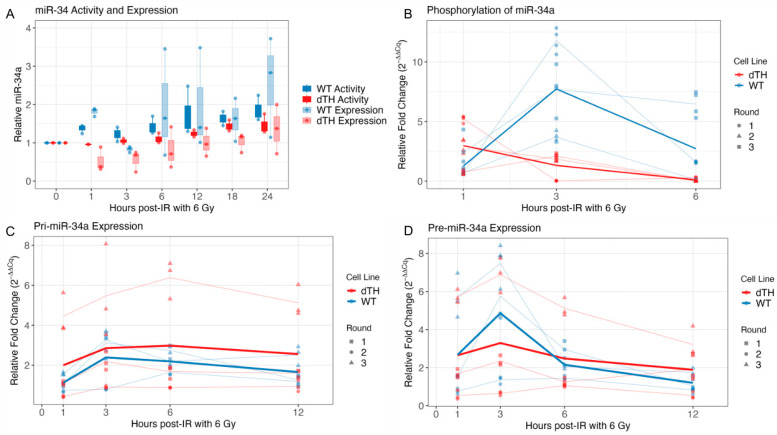
Paraspeckles are associated with the early activity and expression of unphosphorylated miR-34. (**A**) Activity (solid bars, left) and expression (translucent bars, right) of miR-34a over time after a single 6 Gy dose. Activity was calculated by dual luciferase assay and expression by qRT-PCR using 3′ hairpinned primers in WT (blue) and dTH (red) cells. Data represent the mean of three biological replicates, each containing three technical replicates. (**B**) qRT-PCR showing phosphorylated miR-34a levels after IR for WT and dTH A549 cells using a primer ligation method dependent on the presence of a 5′ phosphate. Data represent the mean of three biological replicates, each containing four technical replicates. (**C**) qRT-PCR for miR-34a primary (left) and (**D**) precursor (right) sequences collected in parallel with experiments performed in (**A**).

Consistent with prior observations, in WT cells, we found that the activity of miR-34a after a single 6 Gy dose increased rapidly within 1 h (blue solid bars) prior to a significant increase in expression seen at 6 h (blue translucent bars) detected by qRT-PCR using 3′ hairpin primers (Figure 3A) [13]. In contrast, this early 1 h IR-dependent increase in miR-34a activity prior to an increase in expression was not detected in dTH cells (red translucent bars) and was significantly decreased compared to WT cells (*t*-test *p* = 0.025, Supplemental Table S1C). As expected, both WT and dTH cells demonstrated an increase in miR-34a activity beginning 12 h and later after IR (Figure 3A), consistent with p53-dependent de novo transcription and processing of miR-34a and in agreement with our prior findings [11,13]. Linear mixed-effects modeling confirmed a significant overall increase in activity over time (*p* = 2.4 × 10^−7^), but found no significant main effect of cell line or interaction between time and cell line, indicating similar miR-34a activity after a different early response in WT and dTH cells (Supplemental Table S1A,C).

In parallel with activity, expression of mature miR-34a also increased primarily after 6 h in response to IR [13], but followed a non-linear trajectory best captured by a natural spline (*p* = 0.020, Supplemental Table S1B) (Figure 3A). Overall, WT cells exhibited significantly higher miR-34a expression compared to dTH cells across all time points (*p* = 0.0237) (Supplemental Table S1B,D). The consistently higher levels of miR-34a in WT cells after IR suggest that paraspeckles may be necessary for the stabilization or creation of mature mir-34a. Interestingly, the lack of a significant interaction between time and cell line suggests that the transcriptional induction after initial IR of miR-34a proceeds similarly in both WT and dTH cells.

Paraspeckle loss did not significantly affect activity or expression of miR-17 and let-7 (Supplemental Figure S2), which were previously shown to lack unphosphorylated pools, suggesting the loss of mir-34a early activity and lower overall levels in cells post-irradiation without paraspeckles may be related to the unphosphorylated pool of mir-34a [13].

In addition, we performed adapter ligation PCR that only detects miRNA with a 5′ phosphate. Levels of 5′P miR-34a increased rapidly 3 h after IR in A549WT cells, consistent with prior evidence for rapid radiation-dependent phosphorylation of a pool of unphosphorylated mir-34a and early activity. Importantly, in contrast, there was no increase in the levels of 5′P miR-34a in dTH cells, supporting our hypothesis that the unphosphorylated pool is either not present in these cells lacking paraspeckles or not undergoing early phosphorylation (Figure 3B).

### 2.4. Paraspeckle Loss Is Associated with Changes in Pri- to Pre-mir34a Processing Dynamics

Since paraspeckles have been implicated in the processing of some pri-miRNA [25], we performed qRT-PCR for pri- and pre-miR-34a post-radiation to compare WT and dTH cells. Modeling pri- or pre-miR-34a expression as independent functions of time and cell line revealed no significant differences between WT and dTH cells. However, when the dependent relationship between pri- and pre-miR-34a expression was modeled directly, a divergence emerged, highlighting that the key difference lies in miRNA processing efficiency from pri- to pre-miR rather than overall expression dynamics (Figure 3C,D).

In both WT and dTH cells, pre-miR-34a levels rose steeply with increasing pri-miR-34a expression (*p* = 9.1 × 10^−8^, Supplemental Table S2), consistent with a nonlinear dependency consistent with efficient pri-to-pre processing. The first spline interaction term was not significant (*p* = 0.31), indicating that the initial, approximately linear component of the pri → pre relationship is shared between WT and dTH cells. As shown in Supplementary Figure S3, this linear phase spans pri-miR-34a fold changes of roughly 0–2, where both cell types exhibit comparable processing dynamics and similar rates of pre-miR-34a accumulation. However, the second spline interaction term was significant (*p* = 0.031), demonstrating that the curvature of the pri → pre relationship diverges between cell lines. Beyond ~2-fold pri-miR-34a induction, WT cells continue to efficiently convert pri-miR-34a into pre-miR-34a, whereas dTH cells showed an early plateau—suggesting a possible bottleneck in miRNA maturation under conditions of elevated pri-miR-34a transcription. This suggests that the pri-miR-34a → pre-miR-34a processing step may be rate-limiting in cells without paraspeckles, consistent with prior reports [25].

### 2.5. Nuclear Localization of miR-34a Is Paraspeckle Dependent

**Figure 4:**
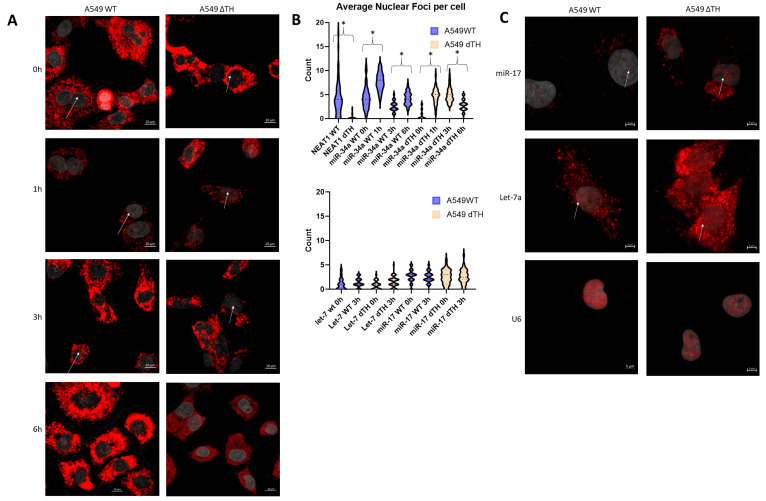
Paraspeckles coordinate punctate nuclear localization (marked by arrows) of miR-34a. (**A**) Single molecule ISH for miR-34a following a single 6 Gy dose in wild-type (left) and dTH (right) cells. (**B**) Quantification of detected nuclear foci for miR-34a (top) or control miRNAs (bottom) in both cell lines over time.(**C**) Puncate nuclear foci do not differ between wild-type and dTH cells for the microRNAs mir-17 and let-7a. Nuclear foci were counted in individual Z stacks—only foci not explained by nearby cytoplasmic signal were considered. In total, 21–56 cells were analyzed for each condition, with an average of 35 cells analyzed. * = *p* < 0.05 by two-sample two-tailed Student’s *t*-test.

To assess the role of paraspeckles in miR-34a nuclear localization, we performed in situ hybridization (ISH) against miR-34a in WT and dTH cells. Prior reports have demonstrated that mature-length unphosphorylated miR-34a resides in the nucleus [13]. Accordingly, ISH against miR-34a in WT cells showed punctate nuclear localization in addition to typical cytoplasmic localization. Nuclear foci were largely abolished in dTH cells lacking paraspeckles (WT mean at baseline = 4.33 vs. dTH = 0.67, *p* = 2.7 × 10^−14^), confirming paraspeckle dependence for baseline nuclear miR-34a (Figure 4A). ISH for miRNA species known to lack an unphosphorylated pool showed occasional nuclear foci that were not affected by deletion of nuclear paraspeckles (Figure 4B,C).

Since IR induces phosphorylation of the unphosphorylated pool and directs its loading onto cytoplasmic Ago2, we expected a loss of punctate nuclear signal in irradiated cells with intact paraspeckles. However, ISH 1 h and 3 h after a single 6 Gy dose showed an increase in nuclear miR-34a foci (Figure 4B). Given that this increase occurs in both WT and dTH A549s (WT: 4.33 → 7.70, *p* = 3.2 × 10^−8^; dTH: 0.67 → 4.71, *p* = 7.6 × 10^−16^) and ISH is not sensitive to 5′ phosphorylation status, we hypothesize that these nuclear foci represent unprocessed pri-miRNAs, as we have seen that there was a similar increase pri-miR-34a in WT and dTH cells above. By 3h post-IR, foci levels declined sharply in WT (7.70 → 2.57, *p* = 3.8 × 10^−13^), consistent with processing/export of pri- to pre-miR34a, while dTH nuclear levels remained elevated (4.71 → 4.92, *p* = 0.60), (Supplemental Table S3A,B) also consistent with possible impaired turnover or altered processing dynamics in the absence of paraspeckles, as shown above of pri- to pre-miR34a.

Other miRNAs investigated in this study are not known to show IR-dependent de novo transcription and likewise did not show an increase in nuclear foci formation after IR (let-7 WT: *p* = 0.31; miR-17 WT: *p* = 0.68, Figure 4B,C). While a nominal increase in nuclear let-7 foci was observed at 1 h (*p* = 0.005), this does not meet Bonferroni-corrected significance after adjusting for 16 comparisons (*p* < 0.003).

## 3. Discussion

In this work, we confirm that unphosphorylated, radiation-responsive miR-34a is localized to the nucleus [13] and demonstrate for the first time that the localization of unphosphorylated miR-34a is dependent on the presence of nuclear paraspeckles. While most mature miRNAs are stabilized by binding to Ago2 concomitant with pre-miRNA processing [36,37], unphosphorylated miRNAs are not Ago2-associated [13], consistent with Ago2 binding directly to the 5′ phosphate [3]. We have demonstrated that loss of paraspeckles reduces early IR-responsive increases in miR-34a activity in dTH cells compared to WT cells. Likewise, 5′ adapter ligation PCR confirms that the increased mir-34a activity in WT cells corresponds with an increase in 5′ phosphorylated miR-34a in cells with intact paraspeckles but not in dTH cells, consistent with prior reports that early miR-34a activity is mediated by rapid phosphorylation of an unphosphorylated pool [13]. We found no difference in post-irradiation pri-miR-34a levels, implying no corresponding changes in de novo transcription in cells lacking paraspeckles. However, we show decreased processing of pri- to pre-mir34a in the dTH cells without paraspeckles, which may explain lower overall mature mir-34a expression post-irradiation in dTH cells compared to WT cells. We have also demonstrated that miR-34a forms punctate nuclear foci in wild-type cells prior to IR, but not in cells lacking nuclear paraspeckles, suggesting that paraspeckles may be necessary for miR-34a nuclear localization. Although ISH does not differentiate between 5′ phosphorylation status, this finding is consistent with the loss of the unphosphorylated mature miR-34a pool we previously found to reside in the nucleus [13].

While this work provides important information on the localization of unphosphorylated miR-34a, further characterization of the mechanism and function of unphosphorylated miR-34a will be valuable. Most notably, while previous work [13] has demonstrated that phosphorylation of the unphosphorylated pool is dependent on the master DNA repair kinase ATM and the nucleotide kinase hClp1, we are not aware of evidence that either protein interacts directly with nuclear paraspeckles or with each other, although ATM co-immunoprecipitates with the paraspeckle protein NUDT21 shortly after IR (Supplemental Figure S1B). Analysis of hClp1 localization is complicated by its lack of punctate nuclear localization both before and after IR. ATM is known to rapidly change localization after DNA damage [38]—studies combining live-cell fluorescence for a paraspeckle marker protein [39] and tagged ATM [40] could potentially reveal ATM-dependent chemistry at the surface of the paraspeckle, which would provide important mechanistic insights not only into activation of unphosphorylated miR-34a, but also of stimulus-specific responses mediated by paraspeckles. To our knowledge, FUS is the only direct ATM target protein resident in nuclear paraspeckles. Future studies of the phosphorylation of paraspeckle-resident FUS by ATM after IR could give important insight into how paraspeckles regulate stress responses and could support a mechanistic model of ATM reaching unphosphorylated miR-34a in paraspeckles.

Our data also do not outline a clear hypothesis for the creation of unphosphorylated miR-34a. While prior work has shown that paraspeckles can regulate processing of specific miRNA species by recruiting Drosha [25], species described in that work do not appear to have unphosphorylated pools, implying that paraspeckle-associated Drosha does not solely produce paraspeckle-associated miRNAs. At baseline, knockdowns of Drosha and Dicer accumulate pri- and pre-miRNAs, respectively, but irradiated WT A549s with these knockdowns still show increased miR-34a activity relative to unirradiated controls 36 h after IR. This may indicate that the unphosphorylated pool is either stable for a prolonged period after transcription and is safely stored in the paraspeckle, or that it is produced independent of canonical miRNA processing [13]. Further investigation into the synthesis rate of unphosphorylated miRNAs, paired with siRNA-mediated inhibition of canonical processing factors, could further reveal if the unphosphorylated pool is synthesized by similar mechanisms used by other miRNAs. Similarly, we do not present a clear mechanism by which miR-34a is regulated within the paraspeckle microenvironment. Previous works have demonstrated sequence-specific, Ago2-dependent binding between NEAT1_2 and miR-34a [23,24], and our data show that a wide range of paraspeckle proteins pull down with biotinylated miR-34a mimics. Future studies aimed at disrupting these binding interactions may highlight more specific mechanisms by which paraspeckles regulate miR-34a.

While future experiments are needed to more fully define the creation of the unphosphorylated miR-34a pool, this work represents an important finding showing associations between nuclear paraspeckles and unphosphorylated miR-34a. Paraspeckles, which have been shown to be critical players in other stress responses, seem to be involved in the localization and early activation of unphosphorylated miR-34a, suggesting coordination of cellular radiation-induced stress responses via maintenance and sequestration of stress-responsive unphosphorylated miRNA pools.

## 4. Materials and Methods

### 4.1. Cell Culture

A549 lung adenocarcinoma cells (ATCC, Manassas, VA, USA) were cultured in F12K media supplemented with 10% fetal bovine serum and 1% streptomycin/penicillin at 37 °C with 5% CO_2_.

### 4.2. Cas9 Editing

Deletion of the NEAT1 triple helix motif was performed by direct lipofection of Cas9 (IDT #1081058, Coralville, Iowa, US) conjugated to Alt-R CRISPR-Cas9 sgRNAs (see Table S1). RNP complexes were assembled in vitro using 1 μM sgRNA and 1 μM Cas9 enzyme and reverse-transfected into 40,000 cells per well for 48 h. Successful editing was confirmed by PCR and sequencing (see Table S1), and loss of paraspeckle formation was confirmed by in situ hybridization against a sequence in the middle of the NEAT1_2 transcript (Stellaris, LCG Biosearch Technologies, Petaluma, CA, USA SMF-2037-1) and immunofluorescence for PSF (Sigma-Aldrich, Burlington, MA, USA P2860).

### 4.3. Mass Spectrometry

A549wt cells were cultured on 10 cm plates in triplicate for each condition as described above. Protein was extracted by scraping plates into 300 μL cold RIPA buffer (Thermo, Waltham, MA, USA #89900). Triplicates were pooled and mixed 1:1 with 2× TENT buffer (20 mM Tris, pH 8.0, 2 mM EDTA, 500 mM NaCl, 1% Triton X-100) with protease inhibitor (Thermo A32965). Lysates were cleared by incubating overnight with 100 μL agarose streptavidin (Vector, Malvern, PA, USA SA-5010). In parallel, 100 μL agarose streptavidin beads were incubated with 600 pmol 3′biotinylated miRNA probe [30] in 1× TENT buffer at 4 degrees Celsius overnight. These miRNA probes consisted of 3′ biotinylated mimics of miR-34a with either a 5′ phosphate or 5′ hydroxyl [41]. Since there exist miRNAs that lack unphosphorylated pools, we also included unconjugated bead controls and a 5′OH 22-nucleotide RNA containing the sequence of miR-34a with the seed sequence inverted to demonstrate sequence specificity of identified proteins. Cleared lysates were incubated with probe-conjugated streptavidin beads in 1× TENT at 4 degrees overnight. Beads were washed three times in 1× TENT without inhibitors and eluted by adding 10 ug RNAse A to each tube for 1 h at room temperature.

ESI-FTICR MS/MS was performed on eluted samples and flowthroughs at the Pasarow Mass Spectrometry facility, UCLA. Candidate proteins were determined by identifying proteins unique to eluates from 5′OH miR-34a 3′biotin-incubated beads with scores above 20 and significant *p*-values. After initial curation, candidate proteins were cross-referenced with RBPDP to identify proteins with canonical RNA-binding domains [31]. Identification of paraspeckle proteins was performed by gene ontology analysis of candidate proteins for cellular components [32,33].

### 4.4. Luciferase Assay

Dual luciferase plasmids were created using the Promega PsiCHECK-2 platform [13]. To create plasmids for measuring the expression of the indicated miRNAs, seed sequences complementary to the target miRNA were edited into the 3′UTR of Renilla luciferase. Briefly, the psiCHECK2 backbone was cut with XhoI and NotI, followed by insertion of oligonucleotides containing the indicated seed sequence. Seed sequences were accessed from miRbase [42], and control sequences were generated using the inverse of the wild-type sequence.

Cells were cultured in 60 mm plates at 50% confluency in triplicate and transfected with 1 ug PsiCHECK-2 plasmid and 5 μL Lipofectamine-2000 per condition. Twelve h after transfection, triplicate plates were pooled and split onto 60 mm plates. Twenty-four h after transfection, cells were irradiated with a single 6 Gy dose at a rate of 1.15 Gy/min.

At the indicated time points, cells were washed twice in PBS and lysed using 750 μL 1× Passive Lysis Buffer (Promega). 20 μL of unclarified lysate was added to a white-walled 96-well plate and measured on a multiwell plate reader (SpectraMax iD3, Molecular Devices, San Jose, CA, USA) in duplicate with a two-second read time and no delay according to the Promega DualGLO protocol (Promega, Madison, WI, USA #E1980).

Relative miRNA activity was calculated according to manufacturer specifications. Briefly, raw luminescence from four readings for each condition was averaged, background luminescence subtracted, and the ratio of luminescence from the Renilla to the firefly luciferase reporter was taken. To assess miRNA-responsive activity, the ratio of parallel transfections of PsiCHECK plasmids containing wild-type miRNA seed sequences or scrambled seed sequences was taken and normalized to unirradiated controls.

### 4.5. RNA Extraction and qRT-PCR

Confluent plates were split onto 60 mm plates at 50% confluency and irradiated with a single 6 Gy dose 24 h after passaging. At the indicated time points after a single 6 Gy dose, plates were scraped into cold PBS, pelleted, and resuspended in 300 μL QiaZOL lysis reagent for RNA purification using the miRNEasy Mini Kit (Qiagen, Venlo, The Netherlands #217004).

Quantification of miRNAs relative to luciferase activity was performed using TaqMan microRNA Assays (Thermo Fisher, #4427975, Waltham, MA, USA), which ligate stem-loop primers onto the 3′ end of target miRNAs. cDNA was synthesized for individual transcripts in parallel using 10 ng of input RNA, and qRT-PCR was performed according to the manufacturer’s instructions. Relative quantifications represent normalization to U6 relative to unirradiated controls collected in parallel.

Quantification of miRNAs with 5′ phosphates was performed using TaqMan Advanced microRNA Assays (Thermo Fisher #A25576). Transcripts were polyadenylated and ligated with 5′phosphate-dependent adaptors according to the manufacturer’s instructions using 10 ng of input RNA per reaction. Reverse transcription was performed according to the manufacturer’s instructions. Relative quantifications represent normalization to miR-17, which was previously shown not to vary in response to ionizing radiation nor contain an unphosphorylated pool [13], relative to unirradiated controls collected in parallel.

Total cDNA for all other transcripts was synthesized according to manufacturer specifications (Thermo Fisher TaqMan miRNA)**.** Briefly, 20 ng of input RNA was mixed 1:1 with a master mix containing random primers to generate whole-genome cDNA for quantification of non-miRNA transcripts. miRNA transcript cDNA was synthesized using transcript-specific hairpin primers—10 ng of input RNA was mixed with 5× TaqMan miRNA probes for each target and master mix. Synthesized cDNA for all targets was quantified using TaqMan amplification on a QuantStudio 3, per manufacturer instructions. Transcript expression was normalized to endogenous U6 expression quantified in parallel. Data represents three independent biological replicates, meaning three separate experiments performed on different days, with three samples each (technical replicates).

### 4.6. Fluorescence Microscopy

22 mm square coverslips (Thermo Fisher #102222) were ethanol-sterilized and coated with poly(L) lysine for 30 min at room temperature, then seeded with 100,000 cells per condition. 24 h after plating, cells were either irradiated for time course experiments or fixed for all other experiments.

miRNA in situ hybridization was performed as previously described [41] with modifications. Briefly, cultured cells were affixed to slides as described above, and desiccation, proteinase K incubation, and acetylation steps were omitted. TSA amplification and hybridization using 2 pmol 5′ DIG-labeled Qiagen miRcury mimics were performed as described, with hybridization at 45 degrees Celsius (miR-34a and let-7) or 50 degrees (miR-17). Streptavidin incubation was performed using 5:100 TexasRed-Streptavidin (Sigma-Aldrich #189738).

NEAT1 ISH and sequential immunofluorescence–ISH were performed using Stellaris FISH probes [43]. Briefly, formaldehyde-fixed coverslips were permeabilized with Triton X-100 and incubated with 1:50 anti-PSF primary antibody (Sigma #P2860) followed by fluorophore-conjugated secondary antibody (Thermo Fisher A32723). Slides were washed and incubated overnight at 37 degrees Celsius with 1 μL 12.5 μM (12.5 pmol) Stellaris FISH probe in 100 μL Stellaris hybridization buffer. Coverslips were washed and mounted on slides with 15 μL VectaShield mounting media with DAPI (Vector Laboratories #H-1000, Newark, CA, USA).

All coverslips were imaged using a Zeiss LSM700 confocal microscope at 100× magnification. Images represent maximum intensity projections of Z-stacks collected on cell nuclei. To ensure verifiable nuclear localization of foci, quantification of nuclear foci was performed on individual slices, with only foci colocalized with DAPI signal and distinct from cytoplasmic signal from neighboring cells were counted. 21–56 cells were counted across three independent replicates for all conditions described.

### 4.7. Statistical Analyses

To compare NEAT1 and NEAT1_2 transcript abundance between wild-type (WT) and deletion of the triple helix (dTH) cell lines, all qRT-PCR values were normalized to their respective WT controls, setting WT expression to 1. Given this normalization removes variance in the WT group, a one-sample *t*-test was performed on the log_2_-transformed relative expression values, which centers the WT reference at 0. This approach allows for symmetric evaluation of up- or down-regulation while preserving the direction and magnitude of change.

To evaluate miR-34a activity and expression over time and compare between WT and dTH, longitudinal measurements were modeled using linear mixed-effects models, with plate number included as a random effect to account for replicate-level variation. Time was modeled as a linear term for activity and as a natural spline (df = 2) for expression to accommodate non-linear trajectories. Additionally, for individual timepoints, pairwise differences between WT and dTH cell lines were assessed using two-sample, two-sided *t*-tests on normalized expression and activity values. Baseline comparisons were excluded as all values at 0 h were set to 1 for normalization.

To assess nuclear foci differences measured by in situ hybridization, two-sample, two-sided *t*-tests were performed. Comparisons were conducted both between WT and dTH cell lines at each time point, and between successive time points within each cell line to evaluate radiation-dependent changes over time. *p*-values were adjusted for multiple testing using a Bonferroni correction across 16 total comparisons, establishing a significance threshold of *p* < 0.003.

We modeled the relationship between *pri*- and *pre*-miR-34a expression following IR using a linear mixed-effects framework. qRT-PCR expression values were first normalized to U6 small RNA expression and then scaled relative to the corresponding unirradiated condition for each cell line at each time point. This normalization strategy captures the log_2_-transformed fold change in expression due specifically to IR, removing baseline differences and technical variation. In the model, *pre*-miR-34a log_2_ fold change was treated as the dependent variable, modeled as a function of cell line (WT vs. dTH), *pri*-miR-34a log_2_ fold change represented by natural spline terms (df = 2) to capture nonlinear processing dynamics, and time post-irradiation (as a linear term). Plate number was included as a random intercept to account for technical variation across replicates. A higher-order spline basis (df = 3) was tested but did not improve interpretability or model fit. The intercept represents *pre*-miR-34a expression in WT cells when *pri*-miR-34a fold change and time are both zero—that is, under baseline (unirradiated) conditions. A statistically significant interaction between cell line and the spline terms indicates that the *pri → pre* processing relationship differs in shape between WT and dTH cells.

## Data Availability

The original contributions presented in this study are included in the article/Supplementary Material. Further inquiries can be directed to the corresponding author.

## Supplementary Materials

The following supporting information can be downloaded at: https://www.mdpi.com/article/10.3390/ncrna12020012/s1. Figure S1: Paraspeckle proteins co-purify with hypothesized interacting factors; Figure S2: Paraspeckle loss does not change activity or expression of other miRs; Figure S3: Relationship between pri-miR-34a and per-miR-34a; Table S1: Statistical analysis of miR-34a activity and expression; Table S2: Fixed effects from mixed-effects model of pre-miR-34a expression as a function of pri-miR-34a expression, time, and cell line after irradiation; Table S3: Statistical comparison of nucelar foci between WT and dTH cells post-irradiation.

## Abbreviations

The following abbreviations are used in this manuscript:

dTH	Deletion of the triple helix A549
miRNA	microRNA
lncRNA	Long-non-coding RNA
ISH	In situ hybridization
ESI-FTICR MS/MS	Electrospray ionization–Fourier transform ion cyclotron resonance tandem mass spectrometry
GO	Gene ontology
df	Degrees of freedom
cDNA	Complementary DNA
RBPDB	RNA-Binding Protein Database
WT	Wild-type A549
qRT-PCR	Quantitative real-time PCR
