# Supplementary material for: Paraspeckles Are Associated with the Activation and Nuclear Localization of Unphosphorylated miR-34a
Source: Noncoding RNA. 2026 Mar 31;12(2):12. doi: 10.3390/ncrna12020012 (PMC13119040; doi:10.3390/ncrna12020012)
Supplement: Supplementary file 1 [file ncrna-12-00012-s001.zip › ncrna-4072959-supplementary.pdf]

Supplemental Data

FIGURES

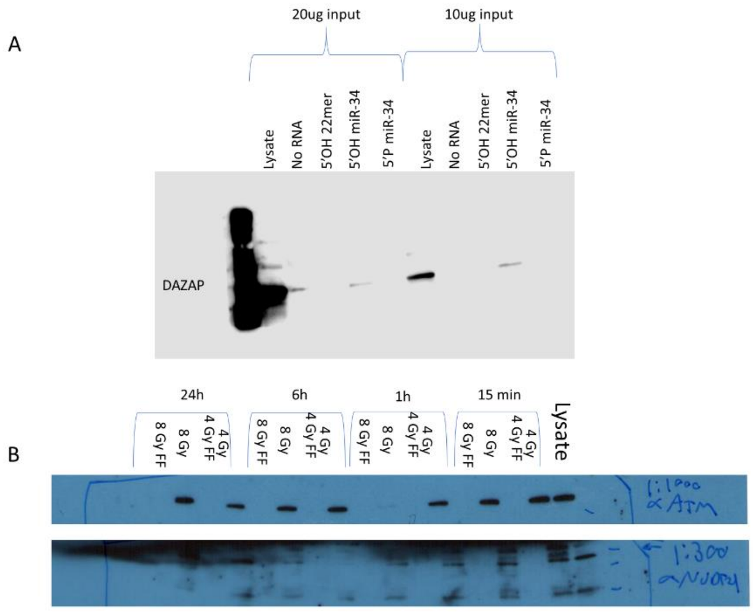

Supplemental Figure S1. Paraspeckle proteins co-purify with hypothesized interacting factors. A) Affinity purification of the indicated miRNA mimic probes with the paraspeckle protein DAZAP. 20 ug (left) or 10 ug (right) of whole-cell lysate were incubated with probes as described above and purified. B) Co-immunoprecipitation of ATM identifies interaction with paraspeckle protein NUDT21 after ionizing radiation. A549wt cells were irradiated and lysed with (FF) or without formaldehyde fixation. Whole cell lysates were incubated with anti-ATM conjugated Protein G agarose beads, purified, and analyzed for presence of NUDT21.

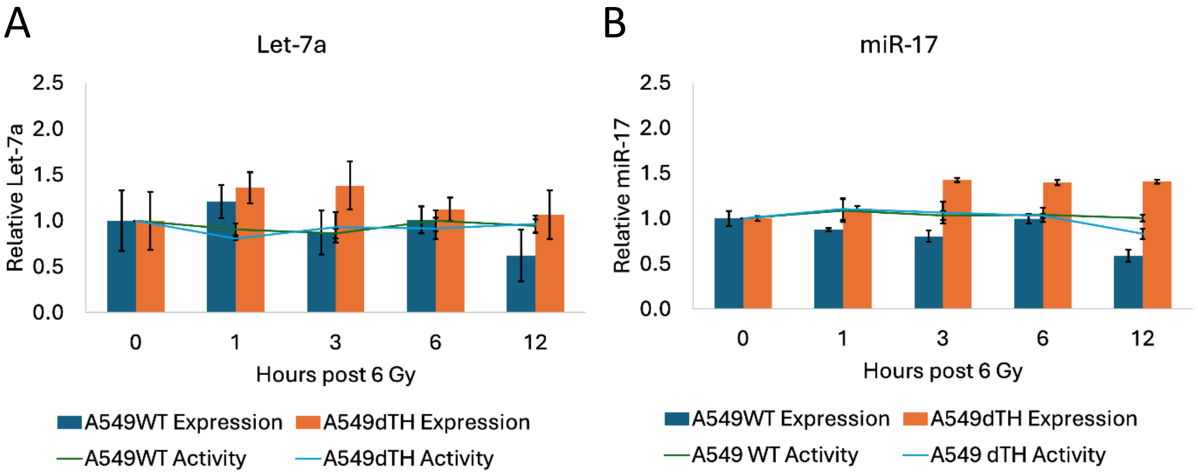

Supplemental Figure S2. Paraspeckle loss does not change activity or expression of other miRs. Paraspeckle loss does not significantly affect activity or expression of let-7a (A) ormiR-17 (B) which were previously shown to lack unphosphorylated pools.

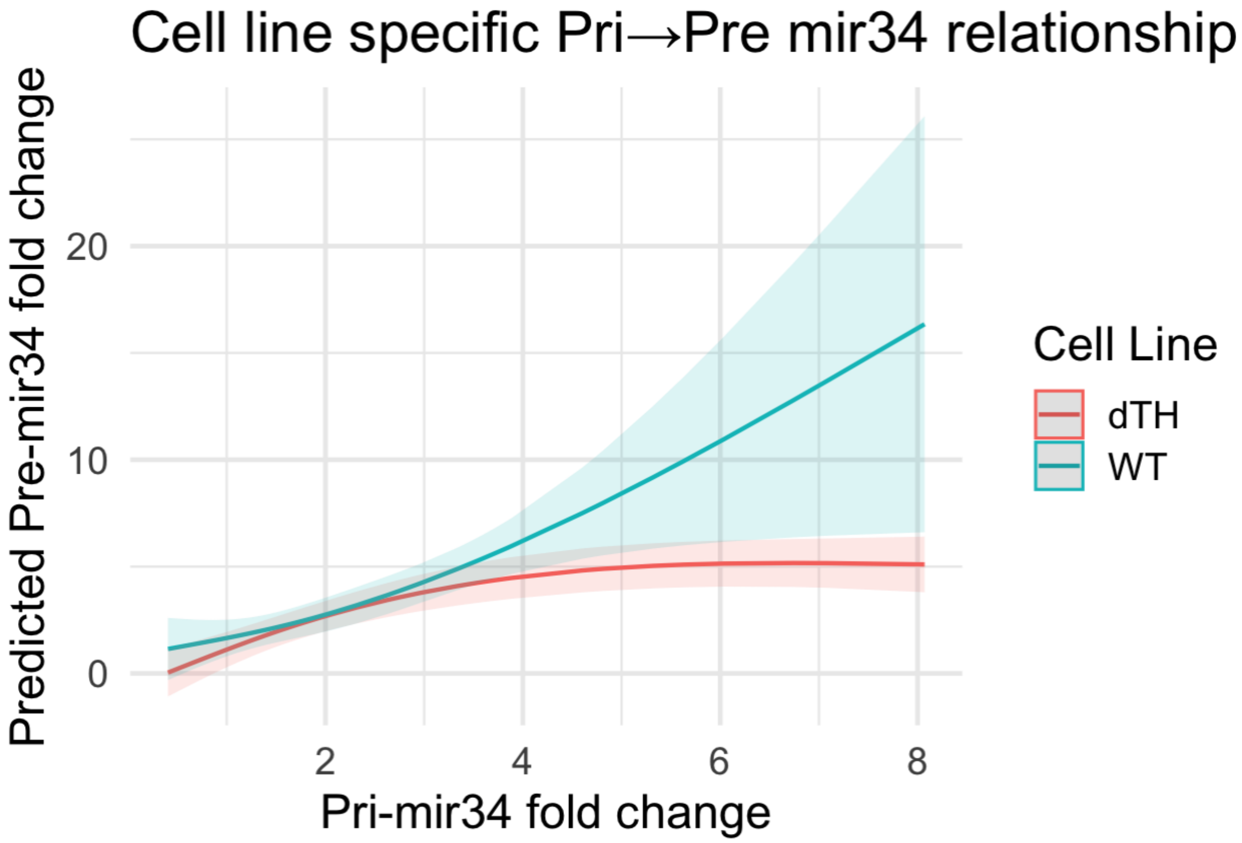

Supplemental Figure S3. Relationship between pri-miR-34a and per-miR-34a. This figure shows the modeled relationship between pri-miR-34 and pre-miR-34 expression levels in wild-type (WT) and dTH cells after irradiation. Each curve represents the predicted fold change in pre-miR-34 for a given level of pri-miR-34 expression, adjusting for time and plate effects. In WT cells (blue), pre-miR-34 levels increase sharply with rising pri-miR-34 expression, indicating efficient processing from pri to pre. In contrast, in dTH cells (red), pre-miR-34 levels plateau even as pri-miR-34 continues to rise, suggesting a defect or bottleneck in pri-miRNA processing. Statistically, this difference is captured by a significant interaction between cell line and the nonlinear spline term (p=0.031), indicating that the *shape* of the relationship differs between cell lines.

Supplemental Table S1. Statistical analysis of miR-34a activity and expression.

Supplemental Table S1A. Fixed Effects for mature mir34a Activity from Mixed Effect Model

	Estimate	SE	df	t value	*p*-value
Time (hrs)	0.031	0.005	31.99	6.52	$2.4{\times}10^{-7}$
Cell Line (dTH vs. WT)	−0.201	0.163	5.36	1.23	0.269
Time x Cell Line	−0.010	0.007	31.99	1.53	0.137

Supplemental Table S1B. Fixed Effects for mature mir34a Expression

	Estimate	SE	df	t value	*p*-value
Spline Time (hrs), component 1	0.750	0.542	34	1.38	0.175

Spline Time (hrs), component 2	0.766	0.312	34	2.45	0.020
Cell Line (dTH vs. WT)	−0.772	0.217	4	−3.55	0.024

Supplemental Table S1C. T-Tests for Activity Differences between WT and dTH

Time (hrs)	WT mean	dTH mean	*p*-value
1	1.37	0.96	0.0247
3	1.22	1.05	0.241
6	1.42	1.11	0.137
12	1.74	1.24	0.307
18	1.64	1.44	0.471
24	1.86	1.46	0.182

Supplemental Table S1D. T-Tests for Expression Differences between WT and dTH

Time (hrs)	WT mean	dTH mean	*p*-value
1	1.81	0.53	0.0122
3	0.83	0.55	0.218
6	1.93	0.83	0.312
12	1.96	1	0.335
18	1.61	1.03	0.207
24	2.56	1.36	0.251

Supplemental Table S2. Fixed effects from mixed-effects model of pre-miR-34a expression as a function of pri-miR-34a expression, time, and cell line after irradiation.

Term	Estimate	Std. Error	t value	*p*-value
Intercept (WT, baseline)	2.1328	0.7623	2.798	0.0082
Cell Line: dTH	−1.1064	0.9147	−1.21	0.234
Pri-miR-34 (spline term 1)	10.8604	1.8022	6.026	9.1×10^{-8}
Pri-miR-34 (spline term 2)	14.3937	4.9726	2.895	0.0054
Time	−0.1797	0.0574	−3.13	0.0058
dTH × Pri-miR-34 (spline term 1)	−2.3914	2.3385	−1.023	0.311
dTH × Pri-miR-34 (spline term 2)	−11.0916	5.0214	−2.209	0.031

Supplemental Table S3. Statistical comparison of nucelar foci between WT and dTH cells post-irradiation.

Supplemental Table S3A. Two-Sample t-tests Comparing Nuclear Foci between WT and dTH

Molecule	Time (hrs)	WT mean	dTH mean	*p*-value	WT n	dTH n
miR-34a	0	4.33	0.67	2.7×10^{-14}	49	36
miR-34a	1	7.70	4.71	3.0×10^{-7}	23	31
miR-34a	3	2.57	4.92	1.1×10^{-8}	21	36
Let-7	0	1.03	0.82	0.44	32	28
Let-7	3	1.30	1.49	0.38	44	47
miR-17	0	2.71	2.55	0.65	31	31
miR-17	3	2.57	2.58	0.99	21	40
NEAT1	0	4.25	0.18	2.6×10^{-11}	56	38

Supplemental Table S3B. Two-Sample t-tests Comparing Nuclear Foci between Subsequent Time Periods

Molecule	Cell Line	Time Comparison	Mean Time1	Mean Time2	n1	n2	*p*-value
miR-34a	WT	0 h vs. 1 h	4.33	7.70	49	23	3.22×10^{-8}
miR-34a	dTH	0 h vs. 1 h	0.67	4.71	36	31	7.56×10^{-16}
miR-34a	WT	1 h vs. 3 h	7.70	2.57	23	21	3.81×10^{-13}
miR-34a	dTH	1 h vs. 3 h	4.71	4.92	31	36	0.600
Let-7	WT	0 h vs. 3 h	1.03	1.30	32	44	0.308
Let-7	dTH	0 h vs. 3 h	0.82	1.49	28	47	5.12×10^{-3}
miR-17	WT	0 h vs. 3 h	2.71	2.57	31	21	0.678
miR-17	dTH	0 h vs. 3 h	2.55	2.58	31	40	0.942
